# Dietary proteins: from evolution to engineering

**DOI:** 10.3389/fnut.2024.1366174

**Published:** 2024-02-16

**Authors:** Hannelore Daniel

**Affiliations:** School of Life Sciences, Technical University of Munich, Freising, Germany

**Keywords:** diet, new proteins, physiology, food biotechnology, evolution

## Abstract

Because of the indispensable amino acids dietary proteins are the most important macronutrients. Proper growth and body maintenance depends on the quantity and quality of protein intake and proteins have thus been most crucial throughout evolution with hominins living in quite diverse food ecosystems. Developments in agriculture and food science have increased availability and diversity of food including protein for a rapidly growing world population while nutrient deficiencies resulting in stunting in children for example have been reduced. Nevertheless, the developing world and growing population needs more protein of high quality – with around 400 million tons *per annum* estimated for 2050. In contrary, protein consumption in all developed countries exceeds meanwhile the recommended intakes considerably with consequences for health and the environment. There is a growing interest in dietary proteins driven by the quest for more sustainable diets and the increasing food demand for a growing world population. This brings new and novel sources such as algae, yeast, insects or bacteria into play in delivering the biomass but also new technologies such as precision fermentation or *in vitro* meat/fish or dairy. What needs to be considered when such new protein sources are explored is that proteins need to provide not only the required amino acids but also functionality in the food produced thereof. This review considers human physiology and metabolism in the context of protein intake from an evolutionary perspective and prospects on future protein production.

## Introduction

Whereas carbohydrates and lipids received over time enormous public interest (sugar tax, low carb, low fat etc.), only recently have proteins experienced a similar public perception. This is fostered by popular press referring to higher protein intakes to help weight loss but also by the wide use of protein supplements in the fitness and bodybuilding scene that often suggest that just eating more protein provides a proper body shape. Markets have seen the emergence of products in almost all food categories with added protein to satisfy this protein-hype. With the high consumption rate of animal-based products and a large array of food items with higher protein contents, the daily protein intake exceeds already the recommended intake levels in all developed countries substantially.

With the awareness of an increasing food demand for the growing world population on background of climate change and expected reductions in agricultural yields and available land area, the interest in proteins from new sources increased drastically. The quest for more sustainable diets in which meat and animal-based food items are replaced has fostered the academic interest in “new proteins” and many research activities have been launched in recent years. These are often embedded into national strategic initiatives to make food systems more resilient and less dependent on imports. While the agri-food sector currently focuses on protein-plants, improved yields and better climate-adopted plant varieties, the biotech sector provides new avenues for food production with renewable energy used to generate hydrogen, acetate or methanol that can serve as energy substrates for biomass production (1, 2). Novel organisms such as chemolithotrophic bacteria have already proven to provide sufficient biomass for protein production by using hydrogen as energy source and by trapping CO_2_ from air (3). Cell culture techniques with stem cells as starters are in development to preplace conventional meat and fish products while “precision fermentation” with recombinant expression of target proteins in production strains of bacteria or yeast is used to provide for example dairy products based on bovine caseins or whey proteins by heterologous expression.

With the framing of “alternative proteins” we are thus exposed to a huge spectrum of approaches and numerous novel technologies that require the assessment of the safety and the nutritional and environmental quality of the new food items. It is the intension of the present review to provide a thoughtful reflection on the role of dietary proteins in human nutrition by taking an evolutionary perspective and by considering the new protein sources as part of future diets.

### Proteins in the evolution of hominins

There can be no doubt that amongst the macronutrients, proteins with the essential amino acids are most crucial diet components in mammalian development. Whereas carbohydrates do not provide any essential entities, lipids have two fatty acids categorized as essential while there are 9 indispensable amino acids. Growth and development require these amino acids but their concentration in food items does not *per se* provide the best spectrum and in particular many plant proteins have individual amino acids in limited quantities. Therefore, combining plant foods to complement the amino acid profiles in the contained proteins is a principle and is realized in many kitchens all over the planet in which a few staple foods are available. This ensures that even with low overall protein contents in the diet optimal conditions for growth and development are realized. In contrast to plants, most animal food sources have amino acid patterns close to those of humans and thus those proteins have played an important role in human development. Although there is a long-lasting discussion on whether hominins evolved as carnivores or vegetarians or even frugivores, it has become obvious that depending on time and geographical location and climate, our ancestors have been consuming whatever the ecosystem provided and that was usually a mix of plant and animal-based products. In this respect it is misleading when argued that there is something as a “paleo-diet.” A new interest in diets of hunters and gatherers emerged in recent years in the biomedical scene when microbiome profiling of stools samples of isolated populations or even of paleosamples revealed that the diversity of gut microbial species was much higher than that found in samples from individuals living in industrialized countries. This is frequently explained with more diverse diets than those of modern times. However, stool analysis of paleosamples (4) but also stool samples collected from people living in rural areas in central Africa (5) showed a high density of parasites and this even correlated with microbiome diversity. This adaptation of bacterial diversity to parasites seems to be a more common phenomenon also in other species (6).

In the Hazda hunter and gatherer society careful analysis of diets – that vary considerably by season – revealed that they have periods in which up to half of their caloric intake comes from honey whereas in other month´ and season up to 65% of daily calorie consumption derives from animal products (7). Such diet patterns we usually consider as most unhealthy but strikingly, Hazda people show low incidence of non-communicable diseases such as cardiovascular diseases (8). But these modern hunter and gatherer groups also provide a good example of how variable human diets have been and can be and how much those depend on the ecosystem setting.

**Figure 1:**
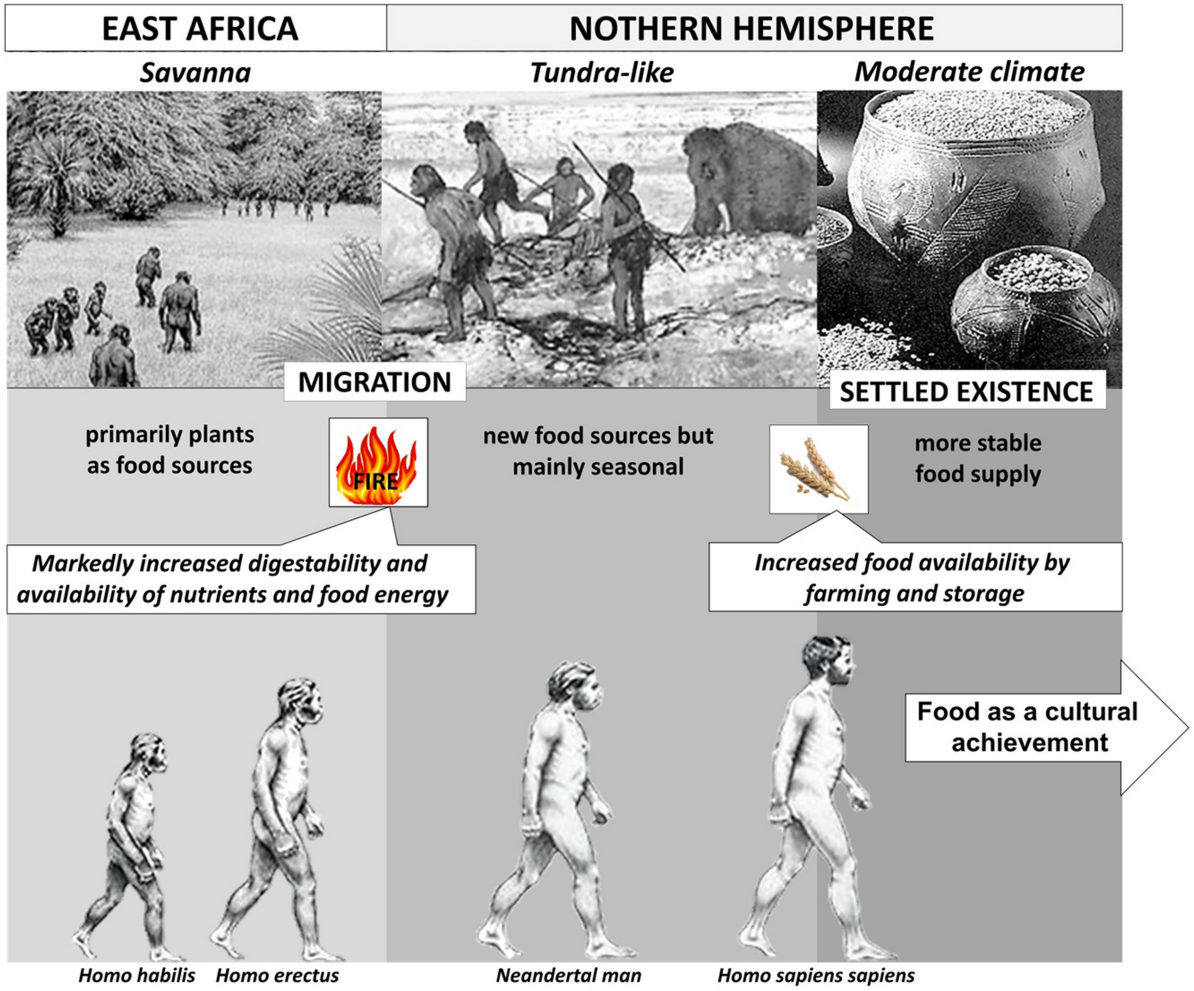
Milestones in the evolution of hominins and the role of diet as derived from different ecosystems.

As a more general finding it has been observed that the percentage of animal products as part of diets increases with a more northern latitude (7). For central and northern Europe that also applies to diets in early hominins. In remains of neanderthal species (age around 40.000 years) and other palaeolithic humans living in Europe, isotope analysis of ^15^N in bone collagen revealed a signature that was even higher than that of pure carnivore species of the same regions suggesting that early humans had a very high rate of animal protein in the respective diets (9). The migration from a rather warm savanna ecosystem into a cold tundra-like landscape in northern Europe and Asia (see Figure 1) was likely associated with a marked change in diet and a high animal protein and fat consumption. The high protein quality of meat was clearly beneficial for development and that was even further enhanced when fire was introduced for food processing estimated as to happened about a million years ago. Raw diets consumed by humans are known to provide insufficient energy and nutrients leading to impairments in the female cycle or even loss of reproductive function (10). Heat treatment of food increases significantly the energy that can be absorbed from the small intestine (11, 12) and this is thought to have changed the anatomy and morphology of the gastrointestinal tract with markedly reduced mass relative to total body mass – leaving more nutrients and energy for brain development and overall body size. With the neolithic revolution and a more constant food supply by farming and animal husbandry, diets may have shifted to a higher proportion of plants while animals still delivered a considerable fraction of protein and fat. Domestication brought new protein sources such as bovine milk into the diet that was driving the adaptaion of humans to intestinal lactase persistance.

Most strikingly, daily protein intake across the world correlates strongly with average male and female body height and even with the phenotype frequency of lactose tolerance (13) suggesting that the high biological quality of dairy protein introduced in the neolithic period also promotes extra growth. Taken together, diets of hominins throughout evolution varied considerably by time, geography, season and ecosystem. Protein supply as well was highly variable but with the indispensable amino acids their contribution of the development of *Homo sapiens sapiens* has been most important for growth and development and similarly, food processing techniques that improved supply and digestibility as well shaped human physiology.

### Proteins in the diet-health relationship

**Figure 2:**
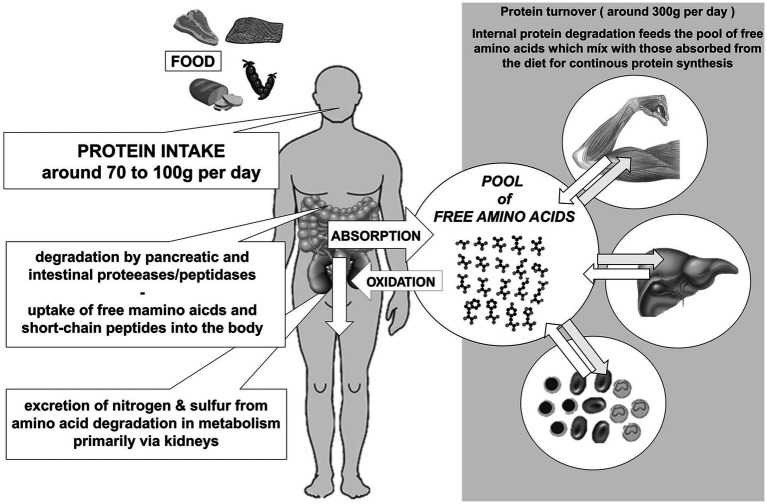
Fate of dietary proteins and turnover of body protein and utilisation of amino acids (per day) in adult humans.

The quantity and quality of dietary proteins has been a key determinant in human development over millennia. Whereas a low protein supply is still a large problem in many low-income countries, daily protein intakes in developed countries exceed meanwhile significantly the recommended intakes (14). In addition, protein quality is no more a critical factor as food availability is unrestricted and with the enormous number of food items – available around the year – and with a high percentage of animal-based product this high protein supply status is just a measure of wealth without a need. Protein intakes are around 90 to 100 g per day in man and around 70 to 75 g per day in woman (14). Required minimum protein intakes when providing high quality proteins to maintain an equilibrium in humans have been estimated to range between 0.3 and 0.5 g/kg body weight/day with the medium requirement defined as 0.66 g leading to a recommended intake of 0.8 or 0.83 g/kg/day as safe population intake in adults and of 1.05 g/kg/day for senior citizens (15). Total protein synthesis in an adult is around 300 g per day with muscle, liver and renewal of circulating blood cells accounting for most of the newly synthesized protein quantities. There is a constant protein breakdown leading to release of free amino acids into a pool in which dietary amino acids enter as well with this pool serving as the resource for *de novo* protein synthesis (see Figure 2). When amino acid intake exceeds the needs, amino acids need to be used as energy substrates leaving nitrogen and sulphur for excretion – mainly via urine.

High protein intakes in high income countries that exceed recommended intake by around 20 to 40 g per individual and day, require that amino acids are immediately oxidised as the capacity for storage of a surplus of amino acids is very limited. Amino acid oxidation leaves nitrogen and sulphur for detoxification which also results in a substantial flux of these entities through the large intestine with effects on the microbiome and not yet adequately defined health consequences (16, 17). Moreover, the surplus of protein with the excess of nitrogen that needs to be excreted has been identified as a problem for the environment (18).

Recently markets have seen a real “protein hype” with many products enriched with an extra portion of protein which further increases total daily protein intake above needed and recommended quantities. This trend is mainly based on the concept that high protein diets reduce caloric intake by a higher satiety signal and thus promote weight loss (19). Indeed, in randomised controlled trials (RCTs) with energy-restricted diets, high protein intake (27–35% of energy intake) provided a greater weight loss (of around 0.8 to 1.2 kg over 1-to-3-month periods) than low protein diets (20). But, meta-analysis of similar RCTs not always confirm such an effect (21) and it seems that as longer the study lasts, as smaller the effects of the high protein diets become (22). In RCTs with non-energy-restricted diets effects on body weight were even smaller with 0.36 kg in >1 month study periods as an example (23) In addition to weight-loss as an endpoint, meta-analysis of many RCTs employing high protein diets could not demonstrate any (21) or only very small beneficial metabolic effects – except for modest reductions in blood triglyceride levels (20, 23). Taken together, long-term effects of high protein diets for improved weight management are scarce and small (19). Benefits of high protein products and diets claimed in the public domain thus often overstate the effect sizes.

Like never before has health become the most crucial criterion in assessing the impact of diets, individual nutrients or even individual food items or any ingredient. In view of the association of protein quantity and protein origin (plant-based or animal product-based) with various disease end-points and all-cause mortality as derived from observational studies it may be concluded that daily protein intakes in the range of up to 30% of energy intake are save with no significant risks for diseases such as cancers or cardiovascular disease (24). If specifically assessed for plant-based or animal-based proteins, it becomes obvious that a high intake of animal protein (up to 20% of energy intake) is associated with a modest increase in disease risks and increased all-cause mortality. This is mainly driven by red meat and processed red meat for which disease-associations have been demonstrated to be dose-dependent (25, 26). Plant-proteins on the contrary appear to have protective effects for most disease endpoints (26).

High protein consumption has for a long time been linked to negative effects on bone-health based on a high production of sulfuric acid when sulphur-containing amino acids are oxidised in metabolism (27, 28). However, recent meta-analysis and umbrella reviews conclude that even with protein intake rates exceeding 1.5 g/kg body weight per day bone mineral density or osteoporosis risk are not impaired – neither in younger nor in older individuals (29). That provides a safe basis for recommendations towards higher protein intakes in senior citizens to prevent or fight sarcopenia (29). Taken together, there is a large body of evidence that higher protein intakes (up to 30% of energy) are safe with some evidence that intake of red meat and processed red meat but not of other meat varieties nor plant proteins increase disease risk (for diabetes, certain cancers, CVD) and all-cause mortality (30).

When we assess environmental footprints of diets or of individual food items, we have an issue with protein intake rates exceeding those recommended. When amino acid intake exceeds the quantity needed for body protein synthesis, amino acids are immediately oxidised and that leaves large quantities of nitrogen for excretion. Urea and ammonia as end-products of this energetic utilisation of the surplus of amino acids are excreted in urine and faeces and this is a burden for the environment. A recent analysis of this nitrogen flow resulting form high protein diets exceeding recommended intake rates estimated for the US (18) a contribution of 28% of all N-emissions into the environment and mainly into the surface water. According to the FAO Statistical Year Book (31) the mean protein supply per day in the US and Europe is similar and N-emissions derived from the surplus of protein intake should therefore be very similar.

### New proteins with new technologies

There can be no doubt that the growing world population needs more protein and that is frequently used as the key argument for initiatives to isolate proteins from any source and with new biomass production approaches employing novel organisms and technologies. But the growing number of humans also need calories and even more so essential micronutrients such as vitamins and trace elements. Based on a model analysis it was concluded that the world produces already enough protein to satisfy the needs of 10 billion people by 2050 – when protein could be distributed evenly (32) whereas micronutrient supply will remain as most critical for billions of people. Iron-deficiency in particular is currently the most important individual nutrient deficiency leading to anaemia in around 500 million woman (31) and providing sufficient dietary iron will remain as one of the key problems in feeding the growing population.

Although there are many new sources for proteins such as micro-and macroalgae like spirulina, chlorella or kelp, mycoproteins, various insect species or new legume varieties (32), none of those sources deliver all essential nutrients that humans need (33). And when used as protein isolates or concentrates, they usually contain also some anti-nutritive compounds such as phytates, protease inhibitors, lectins or other unwanted compounds. Plant-based diets are known to reduce mineral and trace-element bioavailability and that has recently also been raised as concern for the so called “planetary health diet” as a model for future diets with minimal effects on environment but maximal effects on health (34). Moreover, plant-based protein blends (35) or burger patty substitutes (36) were shown to generate lower plasma levels of essential amino acids (EAAS) in postprandial states when provided in identical quantities as the animal-based reference protein. Although human studies in young healthy volunteers ingesting plant-based proteins or protein blends also revealed lower plasma levels of EAAS, differences in post-prandial protein synthesis could not be observed (37). However, in elderly in which postprandial protein synthesis is most important, a lower synthesis rate based on plant-protein rich food may be a problem (35, 38).

That means that when those protein sources are used in food production – mainly for replacing meat or dairy products – essential nutrients such as vitamins, minerals and trace elements need to be added to new food items created. That brings new products into the categories of so called ultraprocessed food (39). The growing consumer demands for plant-based replacers of meat, meat products and dairy has initiated a wide range of activities that go from protein isolation from side-streams or utilisation of new sources such as gras or hemp, to insects, mushroom-mycelium or novel production organisms such as chemo-lithotrophic bacteria.

What is not often considered is the functionality of the protein-concentrates and-isolates (PCIs) when used in production of new food items. Proteins provide many features required in food technology such as solubility, coagulation, emulsification, binding, foaming, gelation or whipping. And most importantly, products based on PCIs need to provide good taste or at least should not bring in off-flavours. Functionality of the various protein preparations is key for market success and that fosters studies that take plant PCIs and test their functional features.

An alternative route could be the synthesis of proteins with a defined functionality via biotechnology. With a better understanding of how amino acid sequence and structure of individual proteins translate into functionality, these proteins can be produced in large scale via expression in yeast – favourably via the methylotrophic yeast *Pichia pastoris* (40). Currently such proteins are produced as modular structures with known functionalities that mimic natural proteins such as collagen. They display interesting technological features such as undergoing temperature-or pH-dependent transitions between liquid and gel states (40). Other examples may be antifreeze-proteins that are employed in certain food categories such as ice-cream (41) or sweet-tasting proteins such as Thaumatin, Brazzein or Monellin (42).

Life science has generated over the last decades functional proteins as tools in cell biology that allow life cell-imaging for example or deliver/capture ligands upon activation by exposure to a defined wavelength of light. Such “protein cages” (43) could as well (given their safety) find numerous applications in food technology but are currently a science fiction domain. However, with the rapid development in artificial intelligence (AI) tools that predict 3D-protein structures from any given sequence and forecasting functional properties, the generation of *in silico* amino acid sequences for *de novo* synthesis of such novel proteins of a defined function may also become an option for food biotech applications of tomorrow.

## Author contributions

HD: Conceptualization, Data curation, Formal analysis, Funding acquisition, Investigation, Methodology, Project administration, Resources, Software, Supervision, Validation, Visualization, Writing – original draft, Writing – review & editing.
